# Gingival hyperplasia as an early manifestation of acute myeloid leukemia. A retrospective review

**DOI:** 10.4317/jced.56214

**Published:** 2019-12-01

**Authors:** Nansi López-Valverde, Antonio López-Valverde, Rafael Gómez-de Diego, Juan M. Ramírez, Javier Flores-Fraile, Jorge Muriel-Fernández

**Affiliations:** 1 Department of Surgery, Instituto de Investigación Biomédica de Salamanca (IBSAL), University of Salamanca, Salamanca, Spain; 2 Department of Oral Medicine. Rey Juan Carlos University, Madrid, Spain; 3 Department of Morphological Sciences. University of Cordoba, Cordoba, Spain

## Abstract

**Background:**

We study the prevalence of acute myeloid leukemia (AML) among patients with severe gingival enlargement.

**Material and Methods:**

We retrospective reviewed the clinical records of patients with severe gingival enlargement, between 2011 and 2018. The Saxer and Mühlemann index were used to measure inflammation and gingival bleeding. The degree of dental mobility was measured by the Nyman and Lindhe technique.

**Results:**

A correlation analysis was carried out to test whether there were any associations among the different variables. In the sample of 117 patients the mean gingival bleeding index was ≥3 and the degree of dental mobility ≥2.3. 1.7% of patients, with severe gingival hyperplasia were diagnosed with AML. We found a significant association between gingival bleeding and aging (*p*<0.001) and a trend (0.54) between bleeding and suffering from AML.

**Conclusions:**

Severe gingival enlargement, abundant gingival bleeding, and dental mobility could be early manifestations of a blood dyscrasia.

** Key words:**Acute myeloblastic leukemia, gingival hyperplasia, bleeding, tooth motility, oral health.

## Introduction

It essential for clinicians to have a sound knowledge of periodontal diseases, not only so that they are able to diagnose this type of pathologies, but also so that they may, in certain cases, learn their real origin when they appear as the first sign of a specific systemic disease.

The new classification scheme for periodontal diseases includes the subsection Hematological Conditions under the section Gingivitis mediated by systemic or local risk factors (1,2).

The current classification associates aggressive periodontitis (AP) with a low bacterial plaque burden but with highly virulent bacteria such as porphyromonas gingivalis, actinobacillus actinomycetemcomitans or tannerella forsythia. The difference between the localized and the generalized forms only refers to the number of affected teeth (3).

Leukemia is a hematological malignancy caused by the proliferation of the tissues that form white blood cells, leading to a sharp increase in circulating immature white blood cells.

Acute myeloblastic leukemia (AML) is the most common type of acute leukemia in adults. The bone marrow is naturally responsible for producing a certain type of cells known as myeloblasts, which mature into granulocytes. In AML, myeloblasts proliferate abnormally, progressively invading the bone marrow and interfering with the production of normal blood cells.

The incidence estimate is 2-3 cases/100,000 per year, gradually increasing with age to a peak of 12.6/100000 in patients aged over 65 years old (4).

In addition to oral ulceration and infection and gingival bleeding, oral and periodontal manifestations of leukemia consist of infiltration of gingivae by leukemic cells, resulting in gingival overgrowth, which develops into pseudo-pockets where dental biofilm accumulates producing, in turn, a second inflammatory lesion that contributes to gum enlargement, so that, gingival enlargement is as much the result of leukemic infiltration as of reactive gingival hyperplasia.

Identifying gingival overgrowth as an initial oral manifestation of leukemic infiltration is extremely important for establishing an early diagnosis of acute leukemia (5). Therefore, with this study our aim is to review the prevalence of acute myeloid leukemia (AML) among patients with severe gingival enlargement treated in a dental clinic.

## Material and Methods

A retrospective review was carried out at the Dental Clinic of the University of Salamanca between 2011 and 2018 (both years included) based on a sample of 16,364 patients, 117 of whom showed a picture of severe gingival hyperplasia associated with spontaneous bleeding and tooth mobility. Of these 117, 62.3% were women and the average age was 59,18.

All the patients underwent periodontal exploration according to a specific protocol, SEPA Code 4 (Spanish Society of Periodontology and Osseointegration), which consisted of a thorough periodontal study (periodontal chart, plaque index, gum bleeding, dental mobility, orthopantomography and an intraoral series of X-rays). In particular those that showed a bag probing greater than 6 mm in 1 or more teeth of a sextant were considered as patients with severe gingival hyperplasia. Inflammation and gingival bleeding indices were measured using Saxer and Mühlemann’s Papillary Bleeding Index (PBI), which has proved very useful in public health programs (6). Tooth mobility was measured through the individual mechanical method, consisting of using two fingers to apply pressure on the crown of the tooth, followed by pressure in the buccolingual direction, registering the degree of tooth mobility according to the technique recommended by Nyman and Lindhe (7).

Patients were later referred to their corresponding primary healthcare centers to be finally examined and assessed by the Hematology Service of the Clinical University Hospital before establishing appropriate periodontal treatment.

Pearson correlation using SPSS© 21.0.0.0 with a statistical significance threshold of *p*<0.05 was used to check correlations among tooth mobility, gum bleeding index, age and suffering from ALM.

Informed consent, formalized at the Dental Clinic, was obtained from all the patients participating in the study and was approved by the Bioethics Committee of the University of Salamanca in accordance with the Helsinki Declaration of 1964.

## Results

The study, based on a large initial sample of 16,364 patients, identified severe gingival hyperplasia in 0.71% (n=117), of whom 1.7% (n=2) were diagnosed with AML. The exploratory data reflected tooth mobility averages of ≥2.4 in women and ≥2.3 in men. The average bleeding index was ≥2.9 in women and ≥3.2 in men. The highest bleeding percentage was observed in women aged ≥62 and in men aged ≥63.  Table 1 and  Table 2 show frequencies base on the determination of the gingival bleeding index and tooth mobility.

**Table 1:**
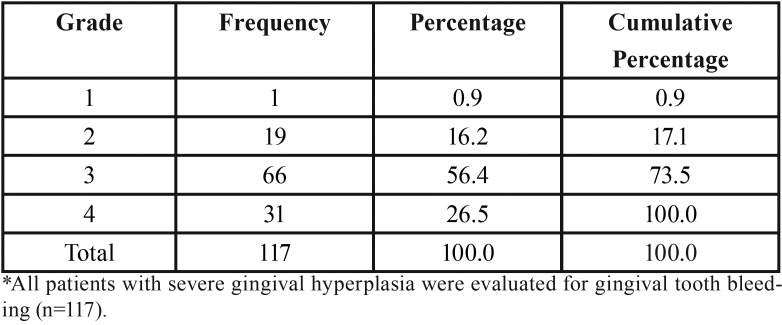
Bleeding index according to Saxer-Mühlemann Index*.

**Table 2:**
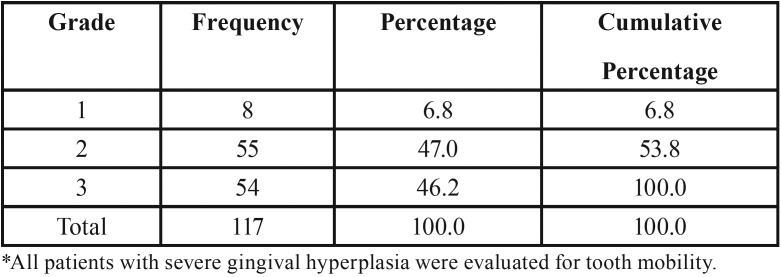
Tooth Mobility according to Nyman-Lindhe index*.

Analyzing the data looking for the prevalence among these patients, the correlation test revealed a highly significant correlation between gingival bleeding index and age, with a Pearson correlation coefficient of 0.361 (*p*<0.001). No significant association was found between age and tooth mobility (Pearson correlation coefficient 0.001, *p*=0.995).

In this study, 1.7 % (n=2) of the patients subjected to the specific protocol established (SEPA Code 4), specifically, a woman and a man with a mean age of 66, were diagnosed with AML. Both patients scored 3 on the bleeding index and were assessed with degree 4 tooth mobility, which are the highest Saxer-Mühlemann and Nyman-Lindhe indexes, respectively.

A correlation test was run to demonstrate the association between the presence or absence of AML and gingival bleeding and tooth mobility degrees. The Pearson correlation coefficient yielded by the analysis was 0.179, with an associated probability of 0.054, which, we believe, shows a trend towards association between high gingival bleeding index scores and suffering from AML. The same test was used to check whether there was any association between suffering from AML and dental mobility, obtaining a statistical figure of 0.131 with an associated *p*-value of 0.160.

## Discussion

To our knowledge, the scientific literature produced to date includes no extensive studies linking gingival hyperplasia to AML, since most of those available refer only to the description of isolated clinical cases. Only Busjan *et al.* (5), in a cross-sectional study, compared the oral mucosa lesions of 39 patients suffering from leukemia with those of 38 healthy patients, observing that 68% of the leukemia patients presented oral mucosa lesions, gingival hyperplasia being this study the most important finding.

Gürkan *et al.*, (8) classify gingival tissue enlargement as hereditary, secondary to prolonged drug therapy (anticonvulsants, antihypertensive drugs, calcium channel-blocking drugs and immunosuppressive drugs) and resulting from different pathological reactions, including under the latter a series of causes that contribute to gingival overgrowth, leukemic cell infiltration among them.

However, we believe that the significance of our findings (Pearson correlation coefficient 0.361, *p*<0.001) could be diminished by the fact that periodontitis is the most common chronic inflammatory condition suffered by people over the age of 65 (60%) and that it usually appears in the form of gum bleeding (9).

Hence, this study highlights that, while there is no significant correlation between gingival bleeding index and suffering from AML, there could be a certain trend between increased bleeding and AML. We believe that even without a significant correlation from a statistical point of view but with a trend (0.054), there is a clinical one that need to be reported, as far as we know, for the first time. Nevertheless, we are aware that, to substantiate this association, it would be necessary to increase the number of cases studied, which would probably require the design of a prospective study.

## Conclusions

Protocolized assessment of gingival hyperplasia by dentists and clinicians, alongside a close collaboration between them and an increase in the number of multicentric collaborative studies could lead, under certain circumstances, to an early diagnosis of AML.
